# Effectiveness of chlorhexidine in preventing infections among patients undergoing cardiac surgeries: a meta-analysis and systematic review

**DOI:** 10.1186/s13756-021-01009-3

**Published:** 2021-10-07

**Authors:** Jianhua Wei, Lingying He, Fengxia Weng, Fangfang Huang, Peng Teng

**Affiliations:** Surgical Intensive Care Unit, The First Affiliated Hospital, College of Medicine, Zhejiang University, Hangzhou, 310003 China

**Keywords:** Chlorhexidine, Cardiac surgery, Wound infection, Surgical complications, Antiseptics, Meta-analysis

## Abstract

**Background:**

Although several meta-analyses reported the impact of chlorhexidine (CHX) use in patients undergoing various types of surgery, no meta-analysis summarized the overall effectiveness of CHX specifically for cardiac surgery. This meta-analysis aimed to examine the impact of CHX on infections after cardiac surgery compared with other cleansers or antiseptics.

**Methods:**

PubMed, Embase, and the Cochrane Library were searched from inception up to October 2020 for potentially eligible studies: (1) population: patients who underwent cardiac surgery; (2) intervention or exposure: any type of CHX use in the treatment or exposed group; (3) outcome: number of patients with infections; (4) comparison: placebo or other antiseptic agents; (5) English. The primary outcome was surgical site infection (SSI).

**Results:**

Fourteen studies were included, with 8235 and 6901 patients in the CHX and control groups. CHX was not protective against SSI (OR = 0.77, 95% CI: 0.57–1.04, *P* = 0.090). CHX was protective for superficial wound infection (OR = 0.42, 95% CI: 0.26–0.70, *P* = 0.001), but not with deep wound infection (*P* = 0.509). CHX was not protective against urinary tract of infection (*P* = 0.415) but was protective for bloodstream infection (OR = 0.36, 95% CI: 0.16–0.80, *P* = 0.012), nosocomial infections (OR = 0.55, 95% CI: 0.44–0.69, *P* < 0.001), and pneumonia (OR = 0.26, 95% CI: 0.11–0.61, *P* = 0.002).

**Conclusions:**

In patients undergoing cardiac surgery, CHX does not protect against SSI, deep wound infection, and urinary tract infections but might protect against superficial SSI, bloodstream infection, nosocomial infections, and pneumonia.

**Supplementary Information:**

The online version contains supplementary material available at 10.1186/s13756-021-01009-3.

## Background

Chlorhexidine (CHX) is a cationic bisbiguanide [1] and an antiseptic agent active against Gram-positive and -negative bacteria widely used in clean surgeries [2–5]. CHX binds to the negatively charged bacterial cell wall to disrupt the cell barrier [1]. CHX is bacteriostatic at low concentrations and bactericidal at higher concentrations [1].

In the past decades, CHX has been used to prevent surgical-related infections [6, 7], such as for decontamination of the oropharynx to avoid respiratory tract infection [8] or for gingival health [9], preoperative skin preparation to avoid surgical site infection [10, 11], or disinfection of medical appliances to avoid nosocomial infection [1, 9, 12–14]. CHX bathing is an effective measure in reducing the levels of pathogens on the skin, and it can also prevent catheter colonization and central line-associated bloodstream infection [15]. CHX is associated with reduced postoperative surgical site infections (SSI) compared with povidone-iodine in clean-contaminated surgery [16].

Although several meta-analyses reported the impact of different types of CHX uses for patients undergoing various types of surgery [17–24], no meta-analysis summarized the overall effectiveness of CHX specifically for cardiac surgery. Indeed, cardiac surgery is highly invasive, usually long, and carries a high risk of infection. Infectious complications occur in 5–21% of the patients after cardiac surgery [25, 26]. After cardiac surgery, the risk of superficial wound infection is 0.5%-8%, and deep sternal wound infections occur in 0.4–2.0% of the cases [25, 26]. Infectious complications prolong the hospital stay, increase healthcare costs, and have dismal outcomes [25, 26].

Therefore, this meta-analysis aimed to examine the impact of CHX on infections after cardiac surgery compared with other cleansers or antiseptics.

## Methods

This meta-analysis was conducted according to the Preferred Reporting Items for Systematic Reviews and Meta-Analyses (PRISMA) guidelines [27]. The relevant articles were searched based on the patient, intervention, comparison, outcome (PICO) principle [28], followed by screening based on the eligibility criteria: (1) population: patients who underwent open cardiac surgery, irrespective of the indication; (2) intervention or exposure: any type of application of CHX in the treatment or exposed group; (3) outcome: number of patients with infections (SSI, pneumonia, bloodstream infection, urinary infection, or nosocomial infection); (4) comparison: placebo, other antiseptic agents, or without CHX; (5) full-text article published in English. PubMed, Embase, and the Cochrane Library were searched from inception up to October 2020 for potentially eligible studies using the MeSH terms of “general surgery” AND “chlorhexidine”, as well as relevant key words such as cardiac or cardiovascular. The exact strategies for all three databases are presented in the Additional file. The literature search and selection of the studies were performed independently by two investigators (Fengxia Weng and Lingying He). Any discrepancy was solved by discussion.

### Data extraction

Study characteristics (authors, year of publication, the country where the study was performed, type of the study design, and number, age, and sex of the patients), exposure parameters (method for the application of CHX, density, and frequency of the treatments); primary outcome (SSI), and secondary outcomes (superficial infection, deep wound infection, bloodstream infection, urinary tract infection, nosocomial infection, and pneumonia) were extracted independently by two investigators (Fengxia Weng and Lingying He). Any discrepancy was solved by discussion.

### Quality of the evidence

The level of evidence of all articles was assessed independently by two authors (Fengxia Weng and Lingying He) according to the Cochrane Handbook for randomized controlled trials [29, 30] and the Newcastle–Ottawa Scale (NOS) criteria for observational studies [31]. Discrepancies in the assessment were resolved through discussion until a consensus was reached.

### Statistical analysis

All analyses were performed using STATA SE 14.0 software (StataCorp, College Station, Texas, USA). The results were summarized as odds ratios (ORs) and 95% confidence interval (95% CI). Statistical heterogeneity among the studies was evaluated using Cochran’s Q-test and the I^2^ index. A Q-test *P*-value < 0.10 and I^2^ > 50% indicated high heterogeneity. Considering the different types of agents in the control group and the various CHX regimens among the included studies, the random-effect model was applied for all analyses to avoid an overestimation of the results. Possible publication bias was not evaluated by funnel plots and Egger’s test because the numbers of studies included in each quantitative analysis were less than 10, in which case the funnel plots and Egger’s test could yield misleading results [29].

## Results

### Literature search

**Fig. 1:**
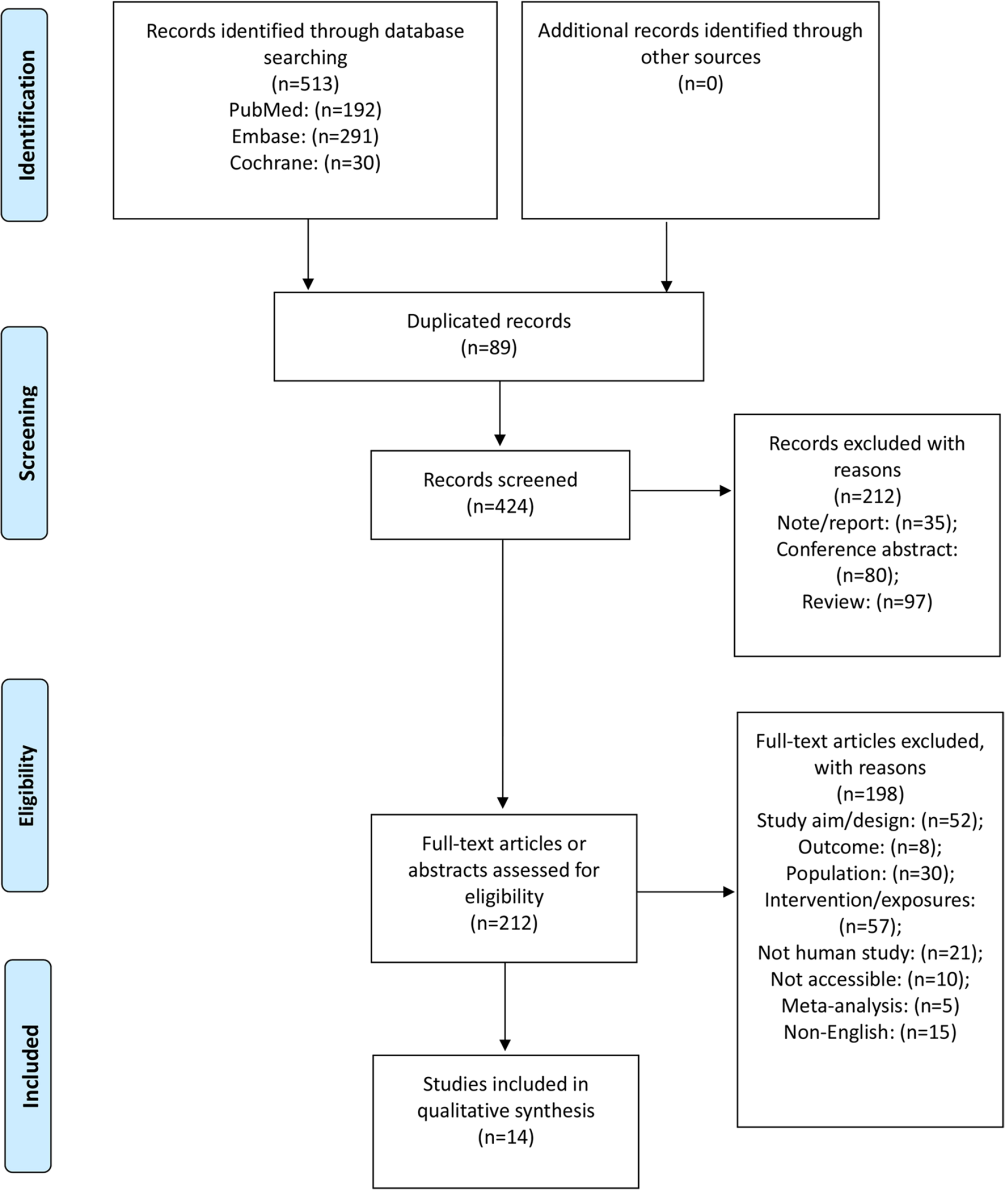
PRISMA 2009 flow diagram

Figure 1 and the Additional file present the literature search process. The initial searched yielded 513 records, and 424 were left after removing the duplicates. These records were screened, and 212 were excluded. The 212 full-text articles or abstracts were assessed for eligibility, and 198 were excluded (52 because of study design/aim, eight for the outcomes, 30 for the populations, 57 for the intervention/exposure, 21 for non-human studies, 10 for no accessible full-text, five for being meta-analyses, and 15 for being published in a language other than English). Finally, 14 studies were included.

### Characteristics of the studies

Among the 14 studies (Table 1), there were five randomized controlled trials [32–36], six prospective cohort studies [37–42], two retrospective cohort studies [43, 44], and one case–control study [45]. Five studies examined the effect of an oral rinse [32–35, 37], four examined skin antiseptic [38–40, 43], and five examined the disinfection of surgical-related appliance [36, 41, 42, 44, 45]. There were 8235 patients in the CHX group and 6901 in the control group.

**Table 1:** Literature search and study characteristic

Author, year	Location	Design	Patients, n		Method of application	Intervention		Frequency of treatment	Age, year	
			CHX	Control		CHX	Control		CHX	Control
**Oral rinse**										
DeRiso [32]	USA	RCT	173	180	Oral rinse	0.12% CHX gluconate	Placebo with comparable color, taste, and smell	2 times a day until ICU discharge	64.1 ± 0.86	63.5 ± 0.84
Houston [33]	USA	RCT	270	291	Oral rinse	0.12% CHX gluconate	\	\	\	\
Segers [34]	USA	RCT	485	469	Oral rinse	0.12% CHX gluconate	Placebo with comparable color, taste, and smell	4 times daily until the day after surgery	65.3 ± 10.4	66.4 ± 9.9
Jacomo [35]	Brazil	RCT	87	73	Oral rinse	0.12% CHX gluconate	Placebo with comparable color, taste and smell	2 times daily until discharge or death	12.2(0–176) months	10.8(0–204) months
Nicolosi [37]	Argentina	Prospective cohort	150	150	Oral rinse	0.12% CHX gluconate	Without CHX	Every 12 h for 3 days	62.3 ± 12.4	63.1 ± 9.3
**Skin antiseptic**										
Hannan [38]	Ireland	Prospective cohort	480	363	Pre-operative Skin antiseptic	2% CHX in 70% alcohol	70% alcohol	\	68 (61–74)	68 (61–75)
Madej [39]	Germany	Prospective cohort	1523	1462	Pre-operative Skin antiseptic	2% CHX combined with 70% IPA	55% IPA	\	67.9 ± 9.9	68.1 ± 9.9
Raja [40]	UK	Prospective cohort	738	738	Pre-operative Skin antiseptic	2% CHX in 70% alcohol	10% IPA	\	\	\
Qintar [43]	USA	Retrospective cohort	1436	1356	Pre-operative skin antiseptic	CHX in alcohol	IPA	\	66 ± 14.14	65.69 ± 15.07
**Disinfection of surgical-related appliances**										
Levy [36]	Israel	RCT	74	71	CHX-impregnated sponge	CHX-impregnated sponge covered with a transparent polyurethane	Transparent polyurethane	\	21 ± 37	31 ± 43
Kohler [41]	Switzerland	Prospective cohort	646	945	Whole-body shower	4% CHX digluconate	Without CHX	Once daily	64.4 ± 13.5	65.2 ± 13.0
Yeo [42]	Korea	Prospective cohort	96	96	Pre-operative disinfection of circuits and hubs	2% CHX + IPA	Without CHX	\	59.4 ± 15.2	56.7 ± 13.4
Abboud [44]	Brazil	Retrospective cohort	82	64	Bathing with CHX-impregnated washcloths	2% CHX	Without CHX	Once-daily	\	\
Thompson [45]	USA	Case–control	1995	643	Bathing with CHX-impregnated washcloths	2% CHX	Without CHX	Once-daily	\	\

*CHX* chlorhexidine, *IPA* isopropyl alcohol

Among the five randomized controlled trials [32–36], only one had an unclear risk of bias for two items [36] (Additional file 1: Table S1). Among the cohort studies [37–44], six studies scored 7 stars [37, 38, 41–44], one scored 8 stars [39], and one scored 9 stars [40] (Additional file 1: Table S2). The case–control study scored 9 stars [45] (Additional file 1: Table S3).

### Surgical site infection

**Fig. 2:**
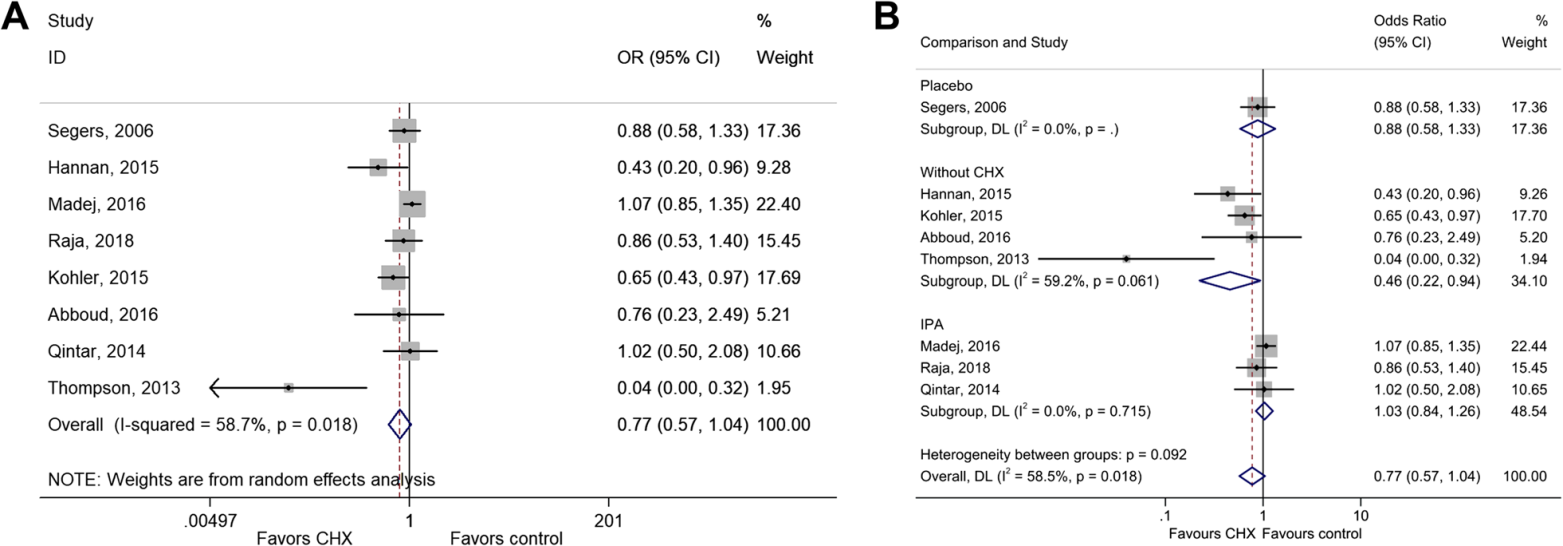
**a** Forest plot of surgical site infection (SSI) comparing patients treated with CHX vs. control (placebo, isopropyl alcohol (IPA), or without CHX). **b** Forest plot of surgical site infection comparing patients treated with CHX vs. control, according to the type of control (placebo, IPA, or without CHX)

Eight studies could be included for the impact of CHX on SSI [34, 38–41, 43, 44]. CHX did not influence the risk of SSI compared with control interventions (OR = 0.77, 95% CI: 0.57–1.04, *P* = 0.090; I^2^ = 63.5%, P_heterogeneity_ = 0.008) (Fig. 2a and Table 2). When considering the type of control, CHX did not influence the risk of SSI compared with placebo or isopropyl alcohol (IPA), CHX protected against SSI when without CHX was used as control (OR = 0.46, 95% CI: 0.22–0.94, *P* = 0.032; I^2^ = 59.2%, P_heterogeneity_ = 0.061) (Fig. 2b).

**Table 2:** Subgroup analyses: CHX vs. control

	N	OR (95% CI)	*P*	I^2^ (%)	P_heterogeneity_
Nosocomial infection	4	0.549 (0.438, 0.687)	< 0.001	0	0.520
Pneumonia	6	0.260 (0.110, 0.611)	0.002	76.6	0.001
Oral rinse	5	0.259 (0.104, 0.643)	0.004	81.2	< 0.001
Disinfection of surgical-related appliances	1	0.257 (0.010, 6.404)	0.407		
Surgical site infection	8	0.757 (0.549, 1.045)	0.090	63.5	0.008
Oral rinse	1	0.881 (0.582,1.333)	0.549		
Skin antiseptic	5	0.824 (0.612, 1.109)	0.201	51.4	0.084
Disinfection of surgical-related appliances	2	0.161 (0.006, 4.395)	0.279	86.9	0.006
Superficial infection	3	0.422 (0.255, 0.696)	0.001	19.4	0.289
Deep infection	3	0.792 (0.397, 1.580)	0.509	51.7	0.126
Bloodstream infection	4	0.362 (0.164, 0.798)	0.012	16.3	0.310
Oral rinse	1	0.256 (0.028, 2.312)	0.225		
Disinfection of surgical-related appliances	3	0.411 (0.138, 1.222)	0.110	43.1	0.173
Urinary tract infection	4	0.795 (0.459, 1.379)	0.415	18.1	0.300
Oral rinse	3	0.746 (0.386, 1.442)	0.384	40.6	0.186
Disinfection of surgical-related appliances	1	1.575 (0.140, 17.766)	0.713		

### Superficial and deep wound infections

**Fig. 3:**
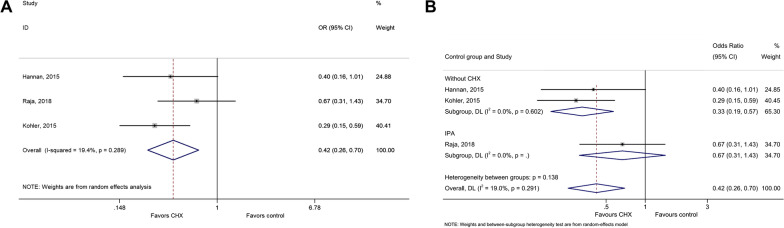
**a** Forest plot of superficial infection comparing patients treated with CHX or control (isopropyl alcohol (IPA) or without CHX). **b** Forest plot of surgical site infection comparing patients treated with CHX vs. control, according to the type of control (IPA or without CHX)

**Fig. 4:**
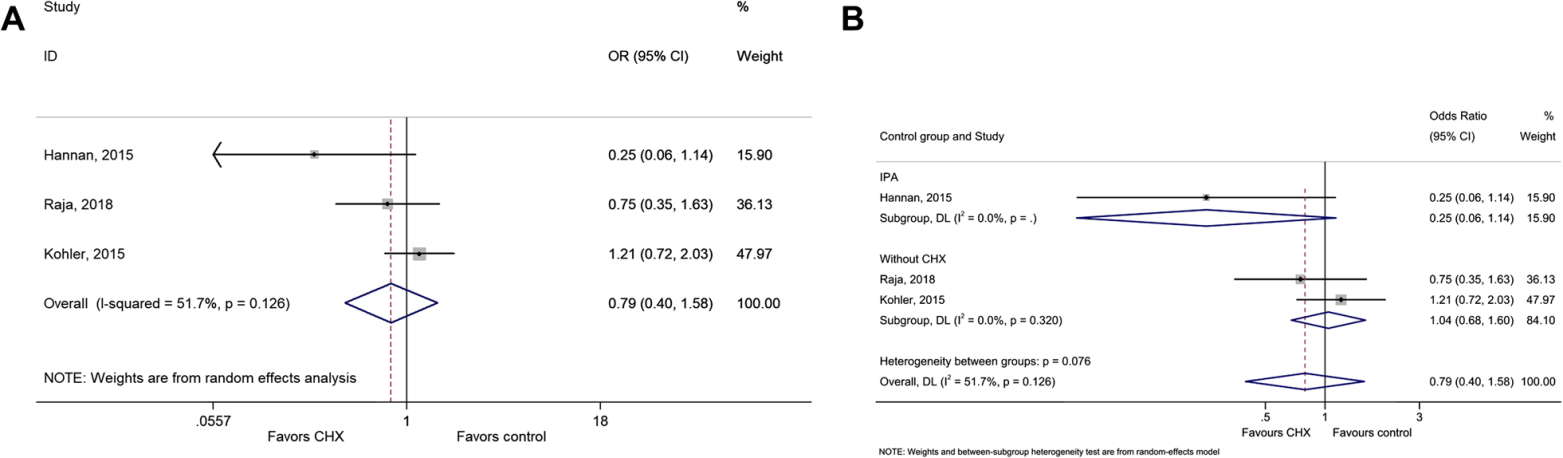
**a** Forest plot of deep wound infection comparing patients treated with CHX or control (isopropyl alcohol (IPA) or without CHX). **b** Forest plot of deep wound infection comparing patients treated with CHX vs. control, according to the type of control (IPA or without CHX)

Three studies could be included to analyze superficial/deep wound infection [38, 40, 41]. CHX was protective against superficial wound infection after cardiac surgery compared with control interventions (OR = 0.42, 95% CI: 0.26–0.70, *P* = 0.001; I^2^ = 19.4%, P_heterogeneity_ = 0.289) (Fig. 3a and Table 2). There was no difference between CHX and IPA, while the difference was driven by no intervention as control (Fig. 3b). No protective effect of CHX was observed for deep wound infection (OR = 0.79, 95% CI: 0.40–1.58, *P* = 0.509, I^2^ = 51.7%, P_heterogeneity_ = 0.126) (Fig. 4a,b and Table 2).

### Effect of the type of CHX intervention

**Fig. 5:**
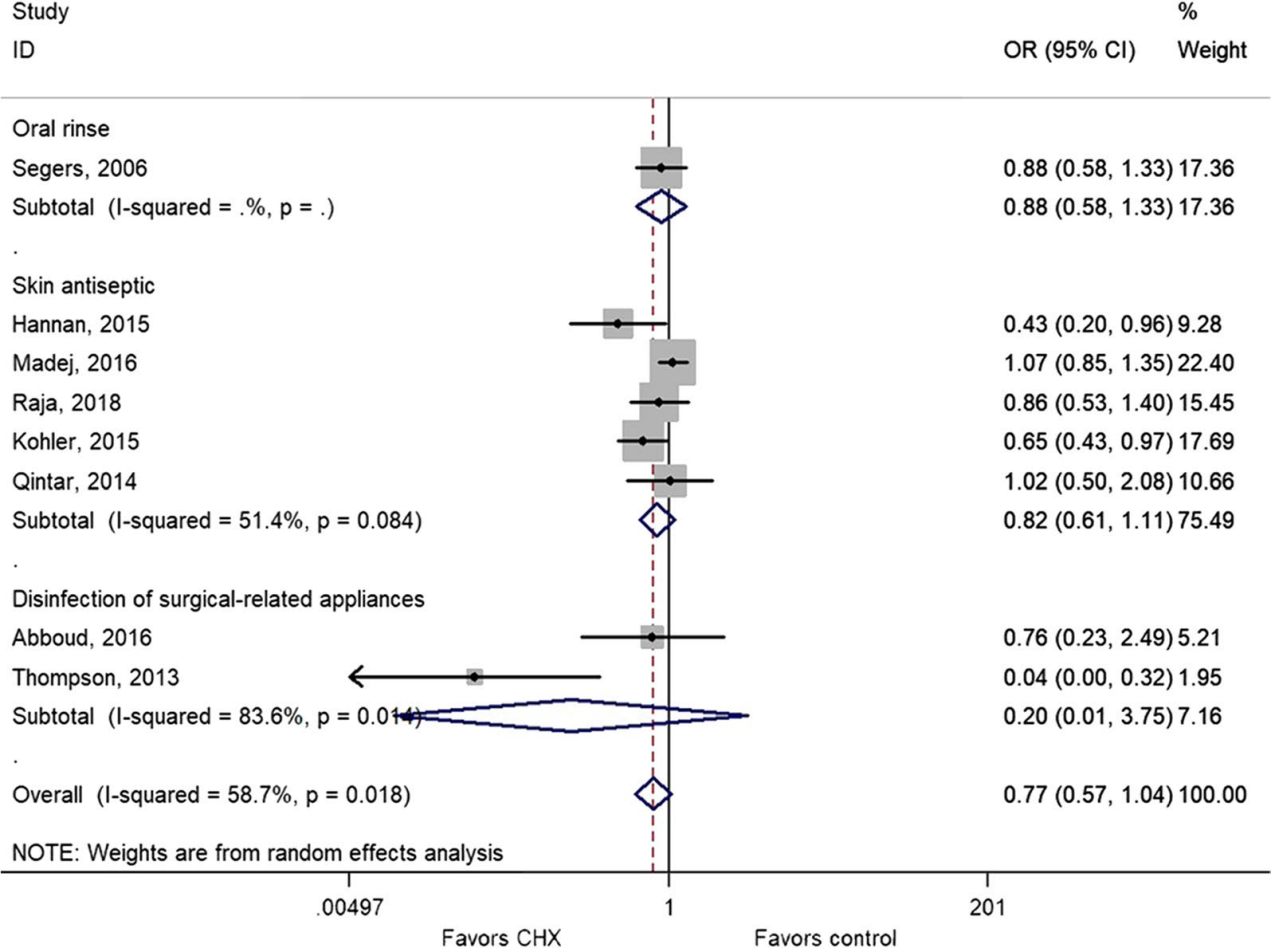
Subgroup analysis on SSI comparing patients treated with CHX or control (IPA or without CHX)

Figure 5 and Table 2 show that the lack of association between CHX and SSI (OR = 0.77, 95% CI: 0.57–1.04, *P* = 0.090; I^2^ = 63.5%, P_heterogeneity_ = 0.008) remained insignificant when considering only the oral rinse (OR = 0.88, 95% CI: 0.58–1.33, *P* = 0.549), skin antiseptic (OR = 0.82, 95% CI: 0.61–1.11, *P* = 0.201; I^2^ = 51.4%, P_heterogeneity_ = 0.084), and disinfection of surgical-related appliance (OR = 0.20, 95% CI: 0.01–3.75, *P* = 0.279; I^2^ = 86.9%, P_heterogeneity_ = 0.006).

### Effect of CHX on infections other than surgical wound

**Fig. 6:**
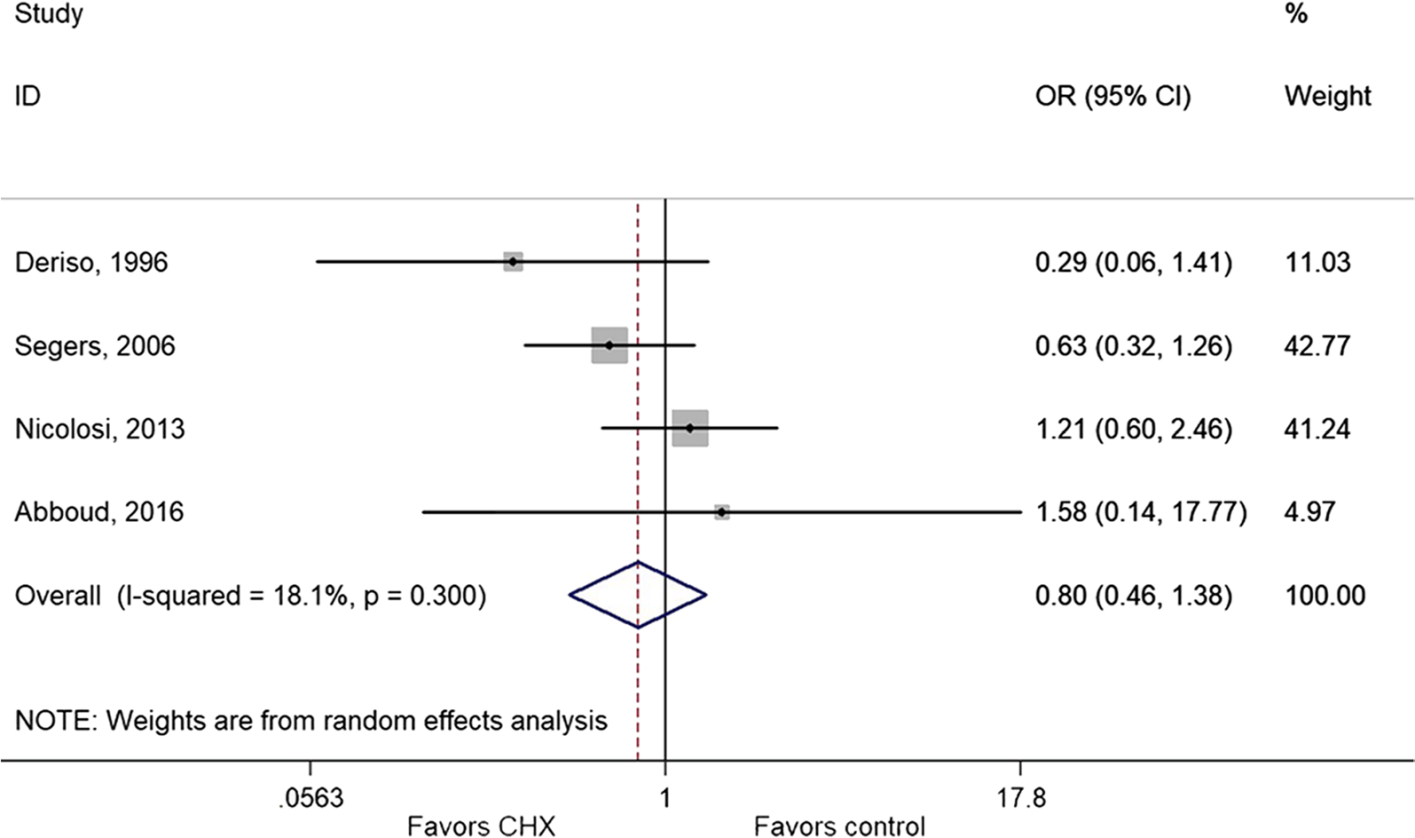
Forest plot of urinary tract infection comparing patients treated with CHX or control (IPA or without CHX)

**Fig. 7:**
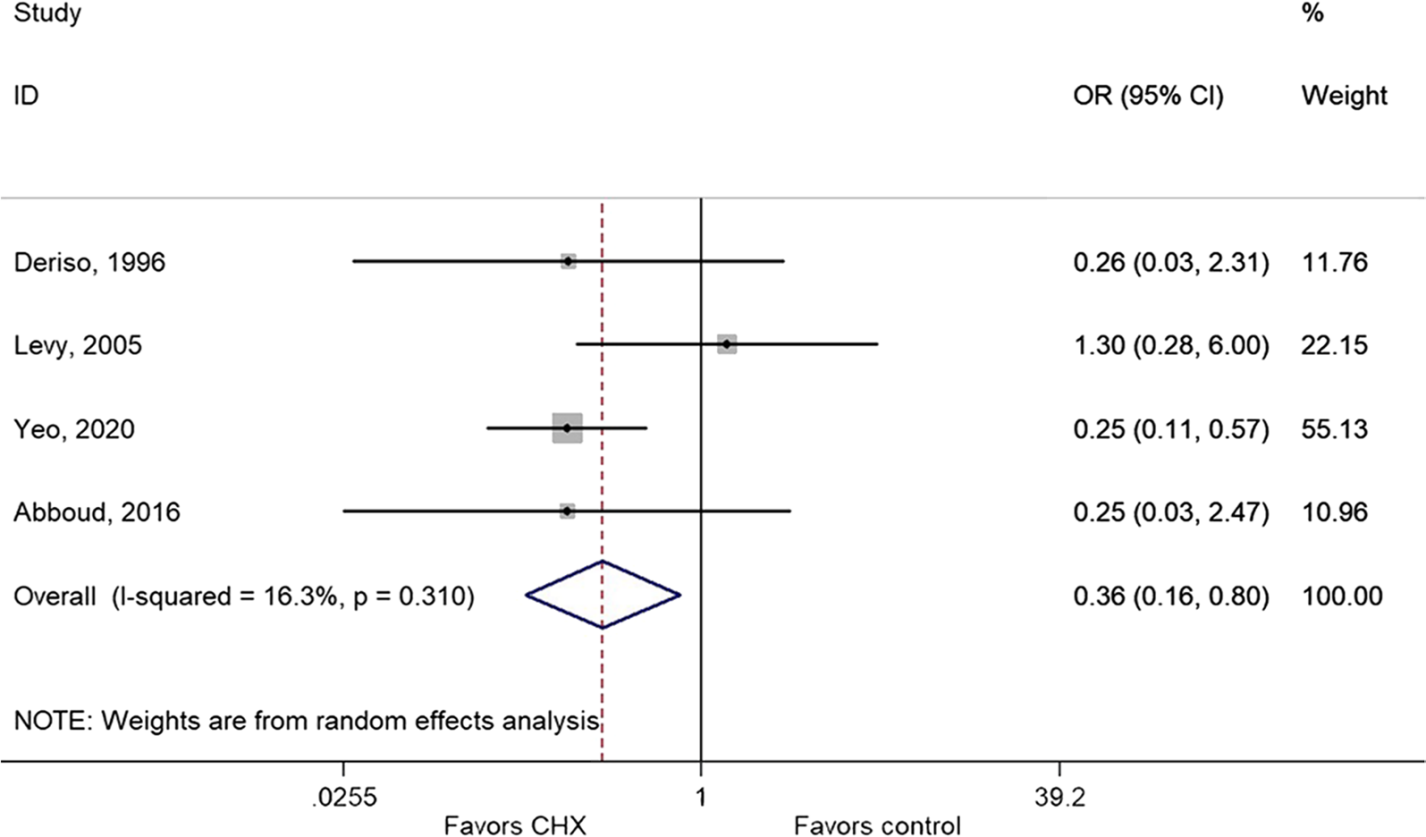
Forest plot of BSI comparing patients treated with CHX or control (IPA or without CHX)

The meta-analysis of four studies [32, 34, 37, 44] showed that CHX was not protective against urinary tract infection (OR = 0.80, 95% CI: 0.46–1.38, *P* = 0.415; I^2^ = 18.1%, P_heterogeneity_ = 0.300) (Fig. 6 and Table 2). On the other hand, the use of CHX protected against bloodstream infection [32, 36, 42, 44] (OR = 0.36, 95% CI: 0.16–0.80, *P* = 0.012; I^2^ = 16.3%, P_heterogeneity_ = 0.310) (Fig. 7 and Table 2), nosocomial infection [32, 34, 35, 37] (OR = 0.55, 95% CI: 0.44–0.69, *P* < 0.001; I^2^ = 0.0%, P_heterogeneity_ = 0.520) (Additional file 2: Fig. S1 and Table 2), and pneumonia [32–35, 37, 44] (OR = 0.26, 95% CI: 0.11–0.61, *P* = 0.002; I^2^ = 76.6%, P_heterogeneity_ = 0.001).

### Sensitivity analyses

Additional file 3: Fig. S3 shows the analyses according to RCTs (A) and observational studies (B). The same conclusions were reached for the two study types for nosocomial infections, pneumonia, SSI, and urinary tract infections. The meta-analysis of RCTs showed no impact of CHX on bloodstream infection, while the meta-analysis of the observational studies showed an impact.

## Discussion

Although several meta-analyses reported the impact of different types of CHX applications for patients undergoing various types of surgery [17–24], no meta-analysis summarized the overall effectiveness of CHX specifically for cardiac surgery, which carries a high risk of infections [25, 26]. Therefore, this study aimed to examine the impact of CHX compared with other cleansers or antiseptics used in cardiac surgeries. The results suggest that CHX was not protective for SSI, deep wound infection, and urinary tract infections but was protective against superficial SSI, bloodstream infection, nosocomial infections, and pneumonia.

The use of CHX before surgery is already well-documented and well-supported by a large amount of evidence, as revealed by many meta-analyses on the subject [17–24]. This meta-analysis adds further evidence by showing that CHX can decrease the risk of superficial SSI, bloodstream infection, nosocomial infections, and pneumonia, leading to poor outcomes in patients who are already affected by their cardiac condition, surgery itself, and often multiple comorbidities. This is supported by other meta-analyses and studies regarding superficial wound infection [12, 40, 41, 46, 47], bloodstream infection [15, 18, 48], nosocomial infections [17, 19, 24, 32–35, 37, 49], and pneumonia [8, 17, 18, 24, 33, 35, 37, 49–51]. Nevertheless, heterogeneity is observed in those previous studies and meta-analyses, mainly due to the different methods of using CHX, the different concentrations, and the different frequencies of use. Nevertheless, the studies agree that CHX generally contributes to the prevention of those infections. On the other hand, the present meta-analysis showed no significant impact of CHX for SSI, which is probably driven by the lack of association with deep wound infection. In addition, there was no association with urinary infections. Nevertheless, previous studies did report associations between CHX and lower odds of those infections [1, 6, 12, 13, 16, 17, 19, 20, 22, 23, 34, 38–41, 44–47, 49]. The discrepancies could be due to several factors such as the included studies, the study populations, and the CHX regimen. Nevertheless, previous meta-analyses that included cardiac surgery patients support the present meta-analysis [18, 24, 51, 52].

Mechanistically, the control of respiratory infection by CHX is probably due to the use of oral CHX [18, 52–56]. Nevertheless, pneumonia can also lead to bloodstream infection [57–59], and preventing one can prevent the other. Regarding the decreased odds of bloodstream infection, this is probably driven by using CHX for the skin and the medical devices since vascular devices are among the first causes of bloodstream infection [60–62]. Of course, decreased superficial SSI is linked to skin disinfection [1, 12, 13, 40, 46]. Future studies could look at the interactions between specific uses of CHX with specific infections, but the present meta-analysis could not perform such analyses.

The conclusions of the meta-analysis must be considered in the light of its limitations. First, we included observational studies in our analysis due to the small number of RCTs in this study field, but at the price of introducing heterogeneity and bias. Second, the treatment regimens in the control group were variable among the studies. Accordingly, the random-effect model was applied to all quantitative analyses regardless of the results of Cochran’s Q test and the I^2^ index. Third, mortality could not be analyzed because it was not reported by enough studies. Finally, despite a relatively large number of patients, the numbers of patients and studies in each subanalysis were small. More studies are required.

## Conclusions

In conclusion, in patients undergoing cardiac surgery, CHX is not protective for SSI, deep wound infection, and urinary tract infections but is protective against superficial infection, bloodstream infection, nosocomial infections, and pneumonia. Therefore, CHX can be useful in cardiac surgeries to prevent infections, especially superficial infection, bloodstream infection, nosocomial infection, and pneumonia. Future studies can work on a standardized protocol to determine the recommended concentration and frequency for different application methods of CHX in patients undergoing cardiac surgery.

## Supplementary Information

**Additional file 1**. Quality of the evidence assessment and the exact strategies for all three databases.

**Additional file 2**.** Figure S1**: Nosocomial infection, comparing patients treated with CHX or control (IPA or without CHX).

**Additional file 3**.** Figure S2**: Pneumonia, comparing patients treated with CHX or control (IPA or without CHX).

**Additional file 4**. ** Figure S3**: Analyses of the outcomes according to randomized controlled trials (A) and observational studies (B).

## Data Availability

The datasets used and/or analysed during the current study are available from the corresponding author on reasonable request.

## Abbreviations

CHX: Chlorhexidine
SSI: Surgical site infection
PRISMA: Preferred Reporting Items for Systematic Reviews and Meta-Analyses
PICO: Patient, intervention, comparison, outcome
BSI: Bloodstream infection
UTI: Urinary tract infection

## References

[1] Bednarek RS, Nassereddin A, Ramsey ML. Skin Antiseptics. StatPearls. Treasure Island (FL) 2020. 29939630

[2] World Health Organization (2009). WHO Model Formulary 2008.

[3] British national formulary : BNF 69 (69 ed.). London: British Medical Association; 2015.

[4] Leikin JB, Paloucek FP. Chlorhexidine gluconate. In: Leikin JB, Paloucek FP, editors. Poisoning and Toxicology Handbook (4th ed.). London: Informa; 2008.

[5] Milstone AM, Passaretti CL, Perl TM (2008). Chlorhexidine: expanding the armamentarium for infection control and prevention. Clin Infect Dis.

[6] Berrios-Torres SI, Umscheid CA, Bratzler DW, Leas B, Stone EC, Kelz RR (2017). Centers for disease control and prevention guideline for the prevention of surgical site infection, 2017. JAMA Surg.

[7] Bratzler DW, Dellinger EP, Olsen KM, Perl TM, Auwaerter PG, Bolon MK (2013). Clinical practice guidelines for antimicrobial prophylaxis in surgery. Am J Health Syst Pharm.

[8] Rello J, Lode H, Cornaglia G, Masterton R, Contributors VAPCB. A European care bundle for prevention of ventilator-associated pneumonia. Intensive Care Med. 2010;36:773–80. 10.1007/s00134-010-1841-5 20237759

[9] James P, Worthington HV, Parnell C, Harding M, Lamont T, Cheung A, et al. Chlorhexidine mouthrinse as an adjunctive treatment for gingival health. Cochrane Database Syst Rev. 2017;3:CD008676. 10.1002/14651858.CD008676.pub2 PMC6464488 28362061

[10] Donskey CJ, Deshpande A (2016). Effect of chlorhexidine bathing in preventing infections and reducing skin burden and environmental contamination: a review of the literature. Am J Infect Control.

[11] Shah HN, Schwartz JL, Luna G, Cullen DL (2016). Bathing with 2% chlorhexidine gluconate: evidence and costs associated with central line-associated bloodstream infections. Crit Care Nurs Q.

[12] Wade RG, Burr NE, McCauley G, Bourke G, Efthimiou O. The comparative efficacy of chlorhexidine gluconate and povidone-iodine antiseptics for the prevention of infection in clean surgery: a systematic review and network meta-analysis. Ann Surg. 2020. 10.1097/SLA.0000000000004076 32773627

[13] Dumville JC, McFarlane E, Edwards P, Lipp A, Holmes A, Liu Z. Preoperative skin antiseptics for preventing surgical wound infections after clean surgery. Cochrane Database Syst Rev. 2015:CD003949. 10.1002/14651858.CD003949.pub4 PMC6485388 25897764

[14] Sinha A, Sazawal S, Pradhan A, Ramji S, Opiyo N. Chlorhexidine skin or cord care for prevention of mortality and infections in neonates. Cochrane Database Syst Rev. 2015:CD007835. 10.1002/14651858.CD007835.pub2 PMC10638659 25739381

[15] Musuuza JS, Guru PK, O'Horo JC, Bongiorno CM, Korobkin MA, Gangnon RE (2019). The impact of chlorhexidine bathing on hospital-acquired bloodstream infections: a systematic review and meta-analysis. BMC Infect Dis.

[16] Noorani A, Rabey N, Walsh SR, Davies RJ (2010). Systematic review and meta-analysis of preoperative antisepsis with chlorhexidine versus povidone-iodine in clean-contaminated surgery. Br J Surg.

[17] Pedersen PU, Larsen P, Hakonsen SJ (2016). The effectiveness of systematic perioperative oral hygiene in reduction of postoperative respiratory tract infections after elective thoracic surgery in adults: a systematic review. JBI Database System Rev Implement Rep.

[18] Silvestri L, Weir WI, Gregori D, Taylor N, Zandstra DF, van Saene JJM (2017). Impact of oral chlorhexidine on bloodstream infection in critically Ill patients: systematic review and meta-analysis of randomized controlled trials. J Cardiothorac Vasc Anesth.

[19] George S, Leasure AR, Horstmanshof D (2016). Effectiveness of decolonization with chlorhexidine and mupirocin in reducing surgical site infections: a systematic review. Dimens Crit Care Nurs.

[20] Lee I, Agarwal RK, Lee BY, Fishman NO, Umscheid CA (2010). Systematic review and cost analysis comparing use of chlorhexidine with use of iodine for preoperative skin antisepsis to prevent surgical site infection. Infect Control Hosp Epidemiol.

[21] Solderer A, Kaufmann M, Hofer D, Wiedemeier D, Attin T, Schmidlin PR (2019). Efficacy of chlorhexidine rinses after periodontal or implant surgery: a systematic review. Clin Oral Investig.

[22] Davies BM, Patel HC (2016). Systematic review and meta-analysis of preoperative antisepsis with combination chlorhexidine and povidone-iodine. Surg J (N Y).

[23] Franco LM, Cota GF, Pinto TS, Ercole FF (2017). Preoperative bathing of the surgical site with chlorhexidine for infection prevention: systematic review with meta-analysis. Am J Infect Control.

[24] Spreadborough P, Lort S, Pasquali S, Popplewell M, Owen A, Kreis I (2016). A systematic review and meta-analysis of perioperative oral decontamination in patients undergoing major elective surgery. Perioper Med (Lond).

[25] Cove ME, Spelman DW, MacLaren G (2012). Infectious complications of cardiac surgery: a clinical review. J Cardiothorac Vasc Anesth.

[26] Gelijns AC, Moskowitz AJ, Acker MA, Argenziano M, Geller NL, Puskas JD (2014). Management practices and major infections after cardiac surgery. J Am Coll Cardiol.

[27] Selcuk AA (2019). A guide for systematic reviews: PRISMA. Turkish Arch Otorhinolaryngol.

[28] Aslam S, Emmanuel P (2010). Formulating a researchable question: a critical step for facilitating good clinical research. Indian J Sex Transmitted Dis AIDS.

[29] Higgins JP, Altman DG, Gotzsche PC, Juni P, Moher D, Oxman AD, et al. The Cochrane Collaboration's tool for assessing risk of bias in randomised trials. Bmj. 2011;343:d5928. 10.1136/bmj.d5928 PMC3196245 22008217

[30] Higgins JPT, Thomas J, Chandler J, Cumpston M, Li T, Page MJ, et al. Cochrane Handbook for Systematic Reviews of Interventions version 6.0 (updated July 2019). London: Cochrane Collaboration; 2019.

[31] Lo CK, Mertz D, Loeb M (2014). Newcastle-Ottawa Scale: comparing reviewers' to authors' assessments. BMC Med Res Methodol.

[32] DeRiso AJ, 2nd, Ladowski JS, Dillon TA, Justice JW, Peterson AC. Chlorhexidine gluconate 0.12% oral rinse reduces the incidence of total nosocomial respiratory infection and nonprophylactic systemic antibiotic use in patients undergoing heart surgery. Chest. 1996;109:1556–61. 10.1378/chest.109.6.1556 8769511

[33] Houston S, Hougland P, Anderson JJ, LaRocco M, Kennedy V, Gentry LO. Effectiveness of 0.12% chlorhexidine gluconate oral rinse in reducing prevalence of nosocomial pneumonia in patients undergoing heart surgery. Am J Crit Care. 2002;11:567–70. 12425407

[34] Segers P, Speekenbrink RG, Ubbink DT, van Ogtrop ML, de Mol BA (2006). Prevention of nosocomial infection in cardiac surgery by decontamination of the nasopharynx and oropharynx with chlorhexidine gluconate: a randomized controlled trial. JAMA.

[35] Jacomo AD, Carmona F, Matsuno AK, Manso PH, Carlotti AP. Effect of oral hygiene with 0.12% chlorhexidine gluconate on the incidence of nosocomial pneumonia in children undergoing cardiac surgery. Infect Control Hosp Epidemiol. 2011;32:591–6. 10.1086/660018 21558772

[36] Levy I, Katz J, Solter E, Samra Z, Vidne B, Birk E (2005). Chlorhexidine-impregnated dressing for prevention of colonization of central venous catheters in infants and children: a randomized controlled study. Pediatr Infect Dis J.

[37] Nicolosi LN, del Carmen Rubio M, Martinez CD, Gonzalez NN, Cruz ME. Effect of oral hygiene and 0.12% chlorhexidine gluconate oral rinse in preventing ventilator-associated pneumonia after cardiovascular surgery. Respir Care. 2014;59:504–9. 10.4187/respcare.02666 24106323

[38] Hannan MM, O'Sullivan KE, Higgins AM, Murphy AM, McCarthy J, Ryan E (2015). The Combined Impact of Surgical Team Education and Chlorhexidine 2% Alcohol on the Reduction of Surgical Site Infection following Cardiac Surgery. Surg Infect (Larchmt).

[39] Madej T, Plotze K, Birkner C, Jatzwauk L, Klaus M, Waldow T (2016). reducing mediastinitis after sternotomy with combined chlorhexidine-isopropyl alcohol skin disinfection: analysis of 3000 Patients. Surg Infect (Larchmt).

[40] Raja SG, Rochon M, Mullins C, Morais C, Kourliouros A, Wishart E (2018). Impact of choice of skin preparation solution in cardiac surgery on rate of surgical site infection: a propensity score matched analysis. J Infect Prev.

[41] Kohler P, Sommerstein R, Schonrath F, Ajdler-Schaffler E, Anagnostopoulos A, Tschirky S (2015). Effect of perioperative mupirocin and antiseptic body wash on infection rate and causative pathogens in patients undergoing cardiac surgery. Am J Infect Control.

[42] Yeo HJ, Kim D, Ha M, Je HG, Kim JS, Cho WH (2020). Chlorhexidine bathing of the exposed circuits in extracorporeal membrane oxygenation: an uncontrolled before-and-after study. Crit Care.

[43] Qintar M, Zardkoohi O, Hammadah M, Hsu A, Wazni O, Wilkoff BL (2015). The impact of changing antiseptic skin preparation agent used for cardiac implantable electronic device (CIED) procedures on the risk of infection. Pacing Clin Electrophysiol.

[44] Abboud CS, de Souza EE, Zandonadi EC, Borges LS, Miglioli L, Monaco FC (2016). Carbapenem-resistant Enterobacteriaceae on a cardiac surgery intensive care unit: successful measures for infection control. J Hosp Infect.

[45] Thompson P, Houston S (2013). Decreasing methicillin-resistant Staphylococcus aureus surgical site infections with chlorhexidine and mupirocin. Am J Infect Control.

[46] Privitera GP, Costa AL, Brusaferro S, Chirletti P, Crosasso P, Massimetti G (2017). Skin antisepsis with chlorhexidine versus iodine for the prevention of surgical site infection: a systematic review and meta-analysis. Am J Infect Control.

[47] Chen S, Chen JW, Guo B, Xu CC (2020). Preoperative antisepsis with chlorhexidine versus povidone-iodine for the prevention of surgical site infection: a systematic review and meta-analysis. World J Surg.

[48] Afonso E, Blot K, Blot S. Prevention of hospital-acquired bloodstream infections through chlorhexidine gluconate-impregnated washcloth bathing in intensive care units: a systematic review and meta-analysis of randomised crossover trials. Euro Surveill. 2016;21. 10.2807/1560-7917.ES.2016.21.46.30400 PMC5144946 27918269

[49] Frost SA, Hou YC, Lombardo L, Metcalfe L, Lynch JM, Hunt L (2018). Evidence for the effectiveness of chlorhexidine bathing and health care-associated infections among adult intensive care patients: a trial sequential meta-analysis. BMC Infect Dis.

[50] Villar CC, Pannuti CM, Nery DM, Morillo CM, Carmona MJ, Romito GA (2016). Effectiveness of intraoral chlorhexidine protocols in the prevention of ventilator-associated pneumonia: meta-analysis and systematic review. Respir Care.

[51] Bardia A, Blitz D, Dai F, Hersey D, Jinadasa S, Tickoo M (2019). Preoperative chlorhexidine mouthwash to reduce pneumonia after cardiac surgery: a systematic review and meta-analysis. J Thorac Cardiovasc Surg.

[52] Klompas M, Speck K, Howell MD, Greene LR, Berenholtz SM (2014). Reappraisal of routine oral care with chlorhexidine gluconate for patients receiving mechanical ventilation: systematic review and meta-analysis. JAMA Intern Med.

[53] Oostdijk EAN, Kesecioglu J, Schultz MJ, Visser CE, de Jonge E, van Essen EHR (2014). Effects of decontamination of the oropharynx and intestinal tract on antibiotic resistance in ICUs: a randomized clinical trial. JAMA.

[54] Melsen WG, de Smet AM, Kluytmans JA, Bonten MJ, Dutch SODSDDTG. Selective decontamination of the oral and digestive tract in surgical versus non-surgical patients in intensive care in a cluster-randomized trial. Br J Surg. 2012;99:232–7. 10.1002/bjs.7703 22021072

[55] Labeau SO, Van de Vyver K, Brusselaers N, Vogelaers D, Blot SI (2011). Prevention of ventilator-associated pneumonia with oral antiseptics: a systematic review and meta-analysis. Lancet Infect Dis.

[56] Shi Z, Xie H, Wang P, Zhang Q, Wu Y, Chen E, et al. Oral hygiene care for critically ill patients to prevent ventilator-associated pneumonia. Cochrane Database Syst Rev. 2013:CD008367. 10.1002/14651858.CD008367.pub2 23939759

[57] Pittet D, Li N, Woolson RF, Wenzel RP (1997). Microbiological factors influencing the outcome of nosocomial bloodstream infections: a 6-year validated, population-based model. Clin Infect Dis.

[58] Agbaht K, Diaz E, Munoz E, Lisboa T, Gomez F, Depuydt PO, et al. Bacteremia in patients with ventilator-associated pneumonia is associated with increased mortality: a study comparing bacteremic vs. nonbacteremic ventilator-associated pneumonia. Crit Care Med. 2007;35:2064–70. 10.1097/01.CCM.0000277042.31524.66 17581489

[59] Sligl W, Taylor G, Brindley PG (2006). Five years of nosocomial Gram-negative bacteremia in a general intensive care unit: epidemiology, antimicrobial susceptibility patterns, and outcomes. Int J Infect Dis.

[60] American Thoracic S, Infectious Diseases Society of A. Guidelines for the management of adults with hospital-acquired, ventilator-associated, and healthcare-associated pneumonia. Am J Respir Crit Care Med. 2005;171:388–416. 10.1164/rccm.200405-644ST 15699079

[61] Masterton RG, Galloway A, French G, Street M, Armstrong J, Brown E (2008). Guidelines for the management of hospital-acquired pneumonia in the UK: report of the working party on hospital-acquired pneumonia of the British Society for Antimicrobial Chemotherapy. J Antimicrob Chemother.

[62] Luna CM, Videla A, Mattera J, Vay C, Famiglietti A, Vujacich P (1999). Blood cultures have limited value in predicting severity of illness and as a diagnostic tool in ventilator-associated pneumonia. Chest.

