# Supplementary material for: Effectiveness of chlorhexidine in preventing infections among patients undergoing cardiac surgeries: a meta-analysis and systematic review
Source: Antimicrob Resist Infect Control. 2021 Oct 7;10:140. doi: 10.1186/s13756-021-01009-3 (PMC8499511; doi:10.1186/s13756-021-01009-3)
Supplement: Supplementary file 1 — Additional file 1. Quality of the evidence assessment and the exact strategies for all three databases. [file 13756_2021_1009_MOESM1_ESM.docx]

**Supplementary Materials**

**1** **Supplementary Tables**

**Supplementary Table 1. Cochrane review for risk of bias**

| Study | Random sequence generation (selection bias) | Allocation concealment (selection bias) | Blinding of participants and personnel (performance bias) | Blinding of outcome assessment (detection bias) | Incomplete outcome data (attrition bias) | Selective reporting (reporting bias) | Other bias |
| --- | --- | --- | --- | --- | --- | --- | --- |
| DeRiso, 1996 (32) | Low | Unclear | Low | Low | Low | Low | Low |
| Houston, 2002 (33) | Low | Low | Low | Low | Low | Low | Low |
| Segers, 2006 (34) | Low | Low | Low | Low | Low | Low | Low |
| Jacomo, 2011 (35) | Low | Low | Low | Low | Low | Low | Low |
| Levy, 2005 (36) | Low | Unclear | Unclear | Low | Low | Low | Low |

**Supplementary Table 2. NOS criteria for cohort study**

| Study | Representativeness of the exposed cohort | Selection of the non-exposed cohort | Ascertainment of exposure | Demonstration that the outcome of interest was not present at the start of the study | Comparability of cohorts on the basis of the design or analysis | Assessment of outcome | Was follow-up long enough for outcomes to occur | Adequacy of follow-up of cohorts | Total quality scores |
| --- | --- | --- | --- | --- | --- | --- | --- | --- | --- |
| Hannan, 2015 (38) | ☆ | \ | ☆ | ☆ | ☆ | ☆ | ☆ | ☆ | 7 |
| Madej, 2016 (39) | ☆ | ☆ | ☆ | ☆ | ☆ | ☆ | ☆ | ☆ | 8 |
| Nicolosi, 2013 (37) | ☆ | \ | ☆ | ☆ | ☆ | ☆ | ☆ | ☆ | 7 |
| Raja, 2018 (40) | ☆ | ☆ | ☆ | ☆ | ☆☆ | ☆ | ☆ | ☆ | 9 |
| Kohler, 2015 (41) | ☆ | \ | ☆ | ☆ | ☆ | ☆ | ☆ | ☆ | 7 |
| Yeo, 2020 (42) | ☆ | \ | ☆ | ☆ | ☆ | ☆ | ☆ | ☆ | 7 |
| Qintar, 2014 (43) | ☆ | \ | ☆ | ☆ | ☆ | ☆ | ☆ | ☆ | 7 |
| Abboud, 2016 (44) | ☆ | \ | ☆ | ☆ | ☆ | ☆ | ☆ | ☆ | 7 |

**Supplementary Table 3. NOS criteria for a case-control study**

| Study | Is the case definition adequate? | Representativeness of the cases | Selection of controls | Definition of controls | Comparability of cases and controls on the basis of the design or analysis | Ascertainment of intervention | Same method of ascertainment for cases and controls | Non-response rate | Total quality scores |
| --- | --- | --- | --- | --- | --- | --- | --- | --- | --- |
| Thompson, 2013 (45) | ☆ | ☆ | ☆ | ☆ | ☆☆ | ☆ | ☆ | ☆ | 9 |

**Supplementary Table 4 Search strategies in PubMed, Embase, and the Cochrane Library**

| ***PubMed*** |  | **Search strategy** | **Numbers** |
| --- | --- | --- | --- |
| **Patient** | **#1** | cardiac OR cordis OR cardiothoracic OR cardiovascular | 2824021 |
|  | **#2** | "General Surgery"[Mesh] OR Surgery, General OR Surgery | 4857077 |
|  | **#3** | #1 AND #2 | 804573 |
| **Intervention** | **#4** | "Chlorhexidine"[Mesh] OR (Chlorhexidine Hydrochloride) OR (Hydrochloride, Chlorhexidine) OR (Tubulicid) OR (Novalsan) OR (Sebidin A) OR (Chlorhexidine Acetate) OR (Acetate, Chlorhexidine) OR (MK-412A) OR (MK 412A) OR (MK412A) | 12517 |
| **ALL** | **#5** | #3 AND #4 | 192 |

| ***Embase*** |  | **Search strategy** | **Numbers** |
| --- | --- | --- | --- |
| **Patient** | **#1** | cardiac OR cordis OR cardiothoracic OR cardiovascular | 2437516 |
|  | **#2** | 'surgery'/exp OR 'diagnosis, surgical' OR 'diagnostic techniques, surgical' OR 'operation' OR 'operation care' OR 'operative intervention' OR 'operative repair' OR 'operative restoration' OR 'operative surgical procedure' OR 'operative treatment' OR 'research surgery' OR 'resection' OR 'specialties, surgical' OR 'surgery, operative' OR 'surgical care' OR 'surgical correction' OR 'surgical exposure' OR 'surgical intervention' OR 'surgical management' OR 'surgical operation' OR 'surgical practice' OR 'surgical procedures, operative' OR 'surgical repair' OR 'surgical research' OR 'surgical restoration' OR 'surgical service' OR 'surgical speciality' OR 'surgical specialty' OR 'surgical therapy' OR 'surgical treatment' | 5555175 |
|  | **#3** | #1 AND #2 | 687287 |
| **Intervention** | **#4** | 'chlorhexidine'/exp OR '1, 1 hexamethylenebis [5 (para chlorophenylbiguanide) ]' OR '1, 1 hexamethylenebis [5 (4 chlorophenyl) biguanide]' OR '1, 6 bis (n5 para chlorophenyl n1 diguanido) hexane' OR '1, 6 bis [n1 (para chlorophenyl) n5 biguanido] hexane' OR '1, 6 di (4 chlorophenyldiguanido) hexane' OR 'ay 5312' OR 'ay5312' OR 'bidex' OR 'boston conditioning lotion' OR 'chlorhex' OR 'chlorhexidin' OR 'chlorhexidine chlorhydrate' OR 'chlorhexidine dihydrochloride' OR 'chlorhexidine glutamate' OR 'chlorhexidine hydrochloride' OR 'chlorohex' OR 'chlorohexidine' OR 'chlorohexidine acetate' OR 'chlorohexydine' OR 'clohexidine' OR 'compound 10040' OR 'hexamethylene 1, 6 bis [1 (5 para chlorophenyl) biguanide]' OR 'lisium' OR 'nibitane' OR 'nolvasan' OR 'nolvascin' OR 'rotersept' OR 'sterilon' OR 'tubilicid' OR 'tubulicid' OR 'umbipro' | 17918 |
| **All** | **#5** | #3 AND #4 | 291 |

| ***Cochrane*** |  | **Search strategy** | **Numbers** |
| --- | --- | --- | --- |
| **Patient** | **#1** | MeSH descriptor: [General Surgery] explode all trees | 351 |
|  | **#2** | (Surgery) OR (Surgery, General) | 236778 |
|  | **#3** | #1 OR #2 | 236778 |
|  | **#4** | cardiac OR cordis OR cardiothoracic OR cardiovascular | 140798 |
|  | **#5** | #3 AND #4 | 30953 |
| **Intervention** | **#6** | MeSH descriptor: [Chlorhexidine] explode all trees | 2217 |
|  | **#7** | (Chlorhexidine Acetate) OR (Acetate, Chlorhexidine) OR (Tubulicid) OR (MK-412A) OR (MK412A) OR (MK 412A) OR (Chlorhexidine Hydrochloride) OR (Hydrochloride, Chlorhexidine) OR (Novalsan) OR (Sebidin A) | 150 |
|  | **#8** | #6 OR #7 | 2311 |
|  | **#9** | #5 AND #8 | 39 (30 trials) |
