# Supplementary figures and images for: Effectiveness of chlorhexidine in preventing infections among patients undergoing cardiac surgeries: a meta-analysis and systematic review
Source: Antimicrob Resist Infect Control. 2021 Oct 7;10:140. doi: 10.1186/s13756-021-01009-3 (PMC8499511; doi:10.1186/s13756-021-01009-3)

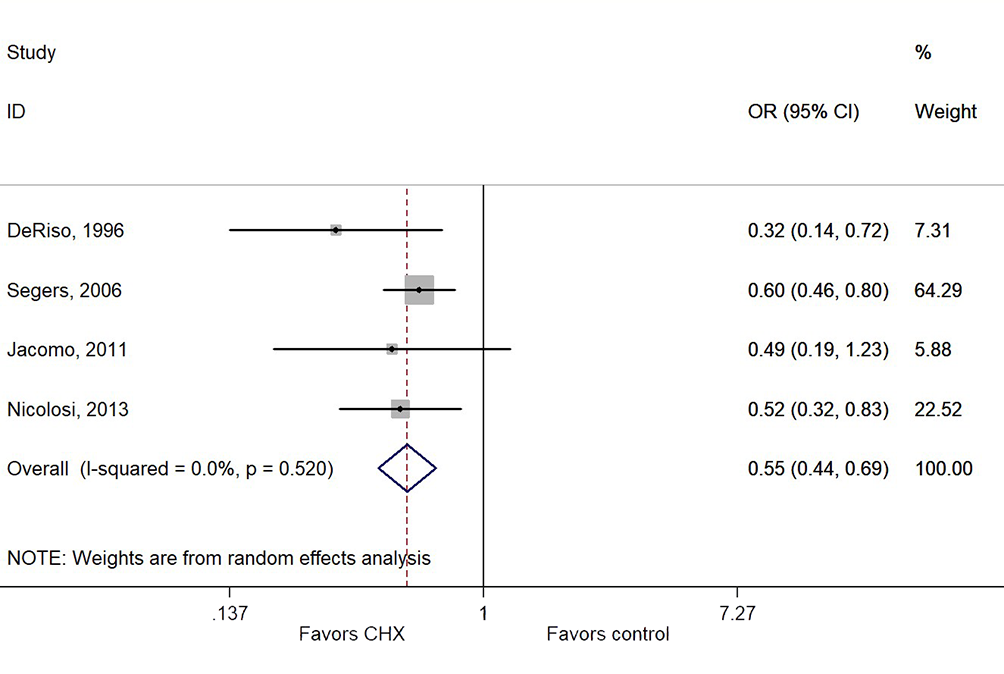

Supplement: Supplementary file 2 — Additional file 2. Figure S1: Nosocomial infection, comparing patients treated with CHX or control (IPA or without CHX). [file 13756_2021_1009_MOESM2_ESM.tif]

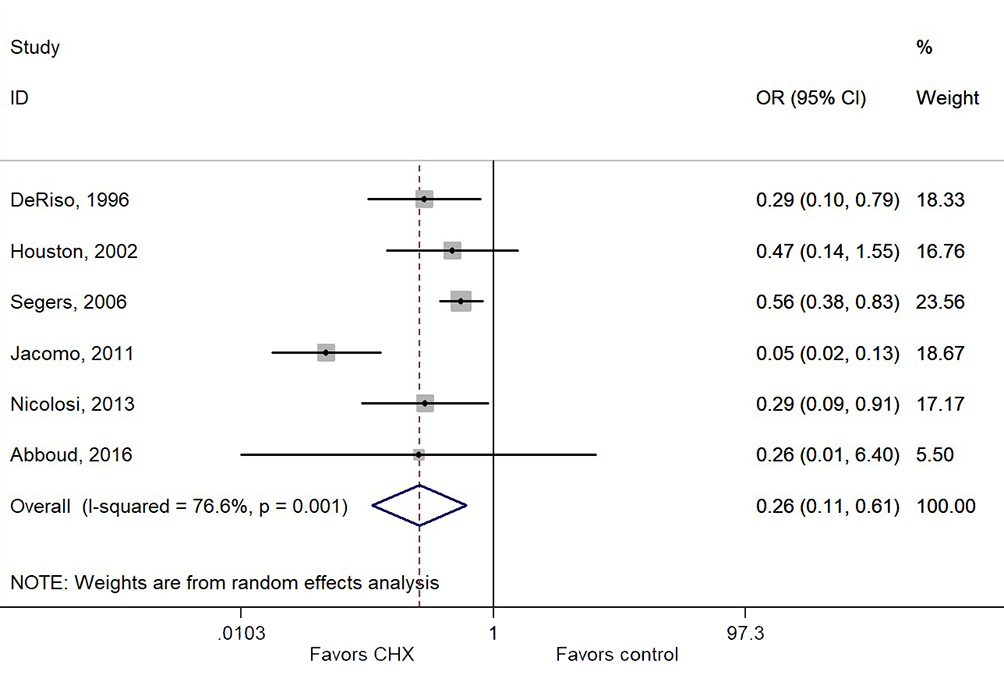

Supplement: Supplementary file 3 — Additional file 3. Figure S2: Pneumonia, comparing patients treated with CHX or control (IPA or without CHX). [file 13756_2021_1009_MOESM3_ESM.tif]

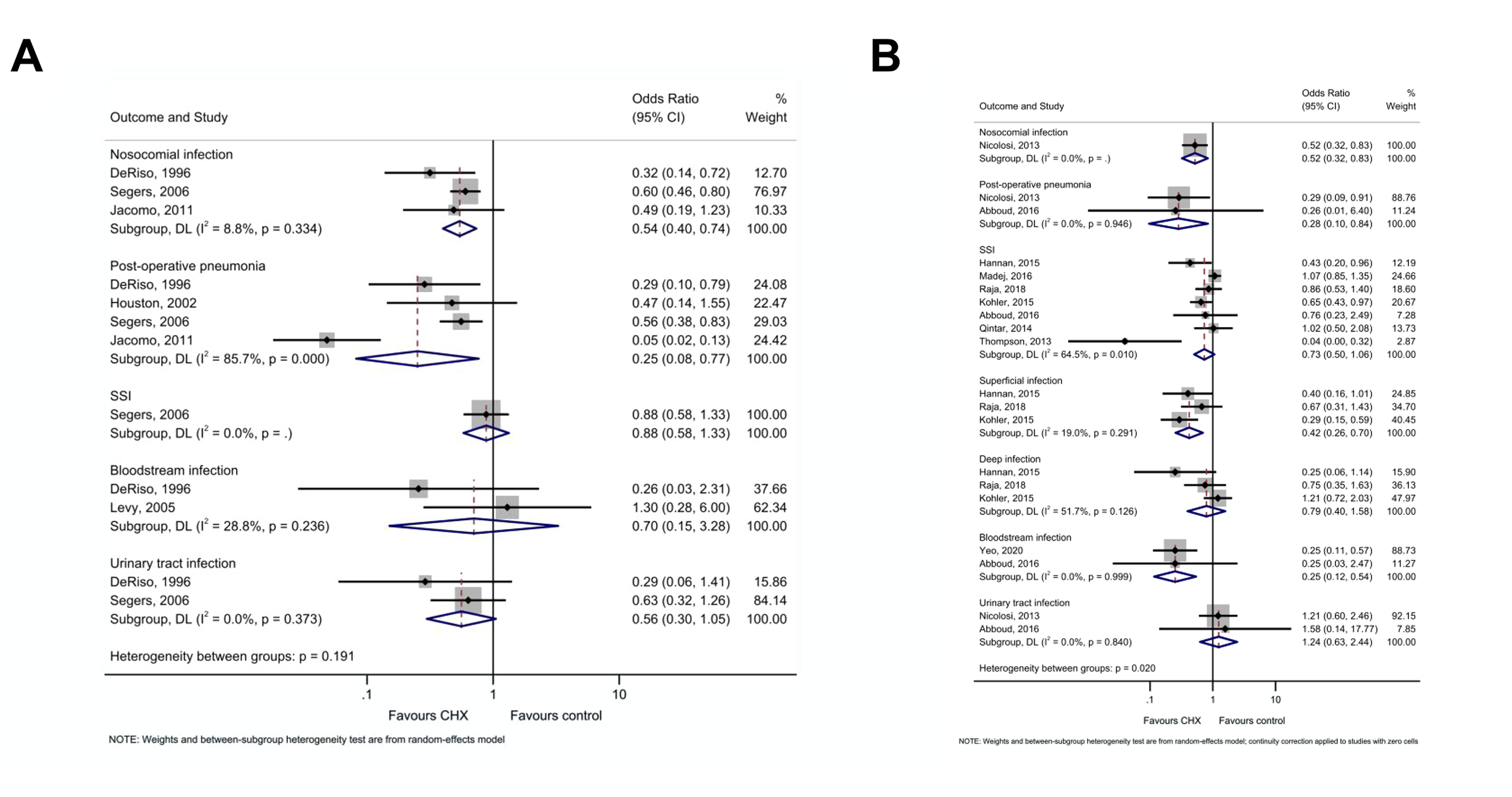

Supplement: Supplementary file 4 — Additional file 4. Figure S3: Analyses of the outcomes according to randomized controlled trials (A) and observational studies (B). [file 13756_2021_1009_MOESM4_ESM.tif]
